# Modeling nursing care tasks in simulated emergency scenarios: insights for clinical training and practice

**DOI:** 10.1038/s44401-026-00079-y

**Published:** 2026-03-09

**Authors:** Nicholas E. Anton, Aditya M. Malusare, Vaneet Aggarwal, Denny Yu

**Affiliations:** Edwardson School of Industrial Engineering, Purdue University, West Lafayette, IN USA

**Keywords:** Computational biology and bioinformatics, Engineering, Health care, Mathematics and computing

## Abstract

Rapid nurse decision-making is needed to detect patient deterioration and prevent mortality. Current approaches to support nurses’ decisions involve diagnostic data processing and providing a decision with little explanation. Our team aimed to demonstrate the utility of attention architecture to model sequential nurse–patient care actions. Experienced nurses and students completed patient care simulations. Nurse actions were systematically coded and analyzed using our model, consisting of an attention encoder to sequentially process and predict nurse behavior. Performance of our model was compared to recurrent neural networks and long-short term memory models based on accuracy, precision, recall, and F1 score. Behavioral data from 24 nurses (11 experienced nurses and 13 nursing students) were collected during patient care simulations. Nineteen unique types of actions were distilled down to 8 common actions. There were 33 episodes captured (i.e., 33 unique sequences of patient care actions), including a total of 1024 actions (i.e., an average of 31 ± 11 actions). Results showed that the attention model outperformed the other models on all metrics except for precision. Our team demonstrated that machine learning can model sequential nurse actions. These results could be leveraged to provide real-time guidance to support novice nurses’ decision-making in the simulated environment.

## Introduction

Among healthcare workers, quick recognition of deteriorating patient status and intervention is needed to prevent patient mortality^1–5^. Given the amount of direct contact they have with patients, and their observation of patient status changes over time, nurses are critical in the recognition of patient deterioration^6^. Recent research investigating the impact of nursing care complexity (i.e., more complex patient care requires more nurse decisions and actions, which were captured by the study team) on patient transfers to more advanced care units has clearly illustrated that higher-acuity patients require more nurse decisions and actions^7^. It is apparent, then, that nurses’ involvement in the patient care process is imperative, particularly for complex patients. However, beyond just identifying the connection between nurse actions and patient complexity, sequentially processing nurses’ patient care actions to effectively care for high complexity patients is also needed.

Experienced nurses make informed decisions by relying on holistic information gathering approaches^8^. Indeed, they often discuss the patient’s status with the patient themselves or family, and utilize physical diagnoses and information from diagnostic equipment (i.e., vital sign monitors) to identify patient care needs and potential risks. However, a systematic review of the literature found that experienced nurses often engage in intuitive and subconscious decision-making processes^9^. Due to the subconscious nature of decision-making processes, nurses may not be cognizant of the factors that impact their decision-making^10^. Furthermore, intuitive decisions are often unable to be recalled or verbalized in real-time what factors contribute to the decision-making process^11^. Thus, it may be difficult to determine which specific experienced nurse actions (i.e., overt actions performed to collect patient information or treat the patient) or sequence of actions contribute to their accurate recognition of patient deterioration and clinical decision making. Real-time continuous modeling of these complex action sequences would be valuable to support inexperienced nurses’ decision-making, as inexperienced nurses are the most susceptible to experience barriers to effective decision-making.

Previous research suggests that experienced nurses are able to leverage their experience caring for a multitude of patients with varying illnesses to resolve typical patient care intuitively encounters or quickly consider patient care protocols to care for rarely encountered patient problems^12^. However, this past research does not adequately detail the entropy and timing of actions performed by experienced nurses when caring for patients. Unlike experienced nurses, novice nurses’ use of intuition in novel clinical situations can lead to diagnostic errors^13^. Novice nurses also do not utilize as many sources of diagnostic information as experienced nurses when making clinical decisions, indicating significant limits to their ability to balance attention among all needed sources of information^14^. Due to their inexperience, novice nurses rely rigidly on patient care protocols to guide their decision making, which increases their confidence in decisions and patient safety^9^. There is a need, then, for novice nurses to follow patient care protocols when faced with unexpected clinical events. However, given that patient status changes are dynamic, myopic focus on performing a standard series of actions may not be optimal. Indeed, despite having protocols available, experienced nurses continue to utilize clinical judgment when determining when to escalate patient care^15^. Recently, researchers have developed a new conceptual framework of nursing work in complex adaptive systems, which indicates that instead of simply considering nurses as executors of actions in a linear manner (e.g., performing prescribed actions according to static protocols), we should consider nurses as active agents who influence and are influenced by dynamics of the system^16^. This framework supports the notion that nurse actions are comprised of intricate patterns that are interdependent. Thus, modeling the intricate patterns of experienced nurse actions may give insights into how nurses care for patients in complex adaptive systems.

Over the past decade, engineers have attempted to develop automated systems to support healthcare workers’ diagnostic accuracy by interpreting patient status trajectories to diagnose illnesses or project future states. A systematic review of the current state of automated nurse decision-making support methods suggests that medical decision support systems can improve care quality, but the results are mixed^17^. The authors argue that these systems, which include methods such as rule-based reasoning and fuzzy logic classifiers to create rule-based decision engines, do not adequately support the nursing care process and feature limited automation to support information gathering^18^. There are also emerging concerns regarding the ability of these “one-size-fits-all” approaches to appropriately capture the nuanced differences in disease presentations of individual patients. Recently, researchers have begun utilizing offline reinforcement learning (RL) approaches to analyze patient trajectories (i.e., compared to historical datasets) to guide medical decision making^19^. In a novel application of offline RL architecture to assist providers in their management of patient complications due to sepsis, researchers implemented a feedback reinforcer to replicate expert provider decisions and identify optimal treatment strategies to enhance patient outcomes^20^. The researchers found that their offline RL decision transformer approach led to very high accuracy when guiding treatment decisions for optimal patient outcomes. However, despite these promising results, there are several potential drawbacks to utilizing these approaches with nurses.

While the approaches to offering healthcare workers decision support can offer numerous advantages for processing individual patient data in the scope of historical data and provider actions, their techniques may not be as salient when healthcare workers must corroborate findings with physical assessments. Ischemic stroke, for instance, involves a rapid increase in a patient’s blood pressure that corresponds with an intravenous blockage that prevents the brain from receiving blood^21^. However, current stroke protocols suggested by the National Institute of Health recommend that nurses perform a series of neurological assessments to verify the symptoms of stroke and rule out other possible diseases^22^. Since clinical decisions cannot be suggested based on vital sign data alone for some disease processes, researchers should consider adapting decision support systems to suggest actions nurses can perform to make informed decisions. This would require, however, modeling approaches that could effectively model sequential nurse action data during the patient care process and accurately predict the next action to be performed.

Attention-based machine learning (ML) approaches are effective for sequence modeling in several ways. Attention architecture is effective for understanding both short- and long-term data patterns^23^. Furthermore, unlike previous models, which struggle with vanishing gradient problems as sequence lengths increase, the attention-based architecture does not suffer from catastrophic forgetfulness^24^. Finally, attention-based models are able to achieve more stable training convergences compared to previous models. Since nursing patient care action sequences may be highly variable, attention architecture may be the most appropriate approach to model patient care actions^25^.

By leveraging the power of attention mechanisms and transformer architecture, the proposed model aims to provide a potential solution for modeling sequences of nursing patient care actions. Accordingly, our team aimed to apply an attention-based encoder to model sequential nursing actions when caring for ischemic stroke patients and demonstrate its effectiveness using a database of experienced nurse action sequences caring for simulated patients suffering from strokes. This approach was compared to other commonly used ML approaches to study their ability to model sequential task data and predict subsequent actions using multiple measures of accuracy. We hypothesized that our approach would be more accurate than other commonly used ML approaches to predict nurses’ patient care actions.

## Results

### Participant demographics

Eleven experienced nurses completed the stroke and Covid-19 scenarios (i.e., nine nurses completed both scenarios, one nurse completed only the stroke case, and one nurse completed only the Covid-19 scenario), and 13 nursing students completed the stroke scenario. Participating experienced nurses had significant clinical experience at the time of the study. Twenty-seven percent of nurses were 30–39 years old, and 27% were 40–49 years old. The average post-training clinical experience of nurses was 15.3 years. All experienced nurses had obtained a Bachelor's of Science in Nursing, while 27% of nurses had obtained a Master's of Science in Nursing. Nursing students were all in the 3rd year of their undergraduate degree at the time of the study.

### Nurse action data

**Fig. 1:**
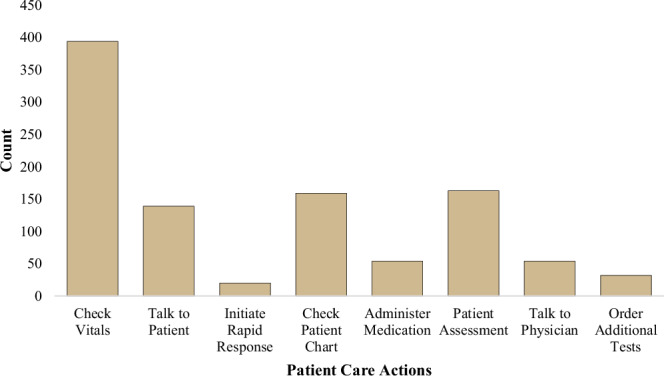
Distribution of actions performed. Our cumulative dataset consists of sequences of actions categorized into eight labels, with the distribution as shown in the above figure.

**Fig. 2:**
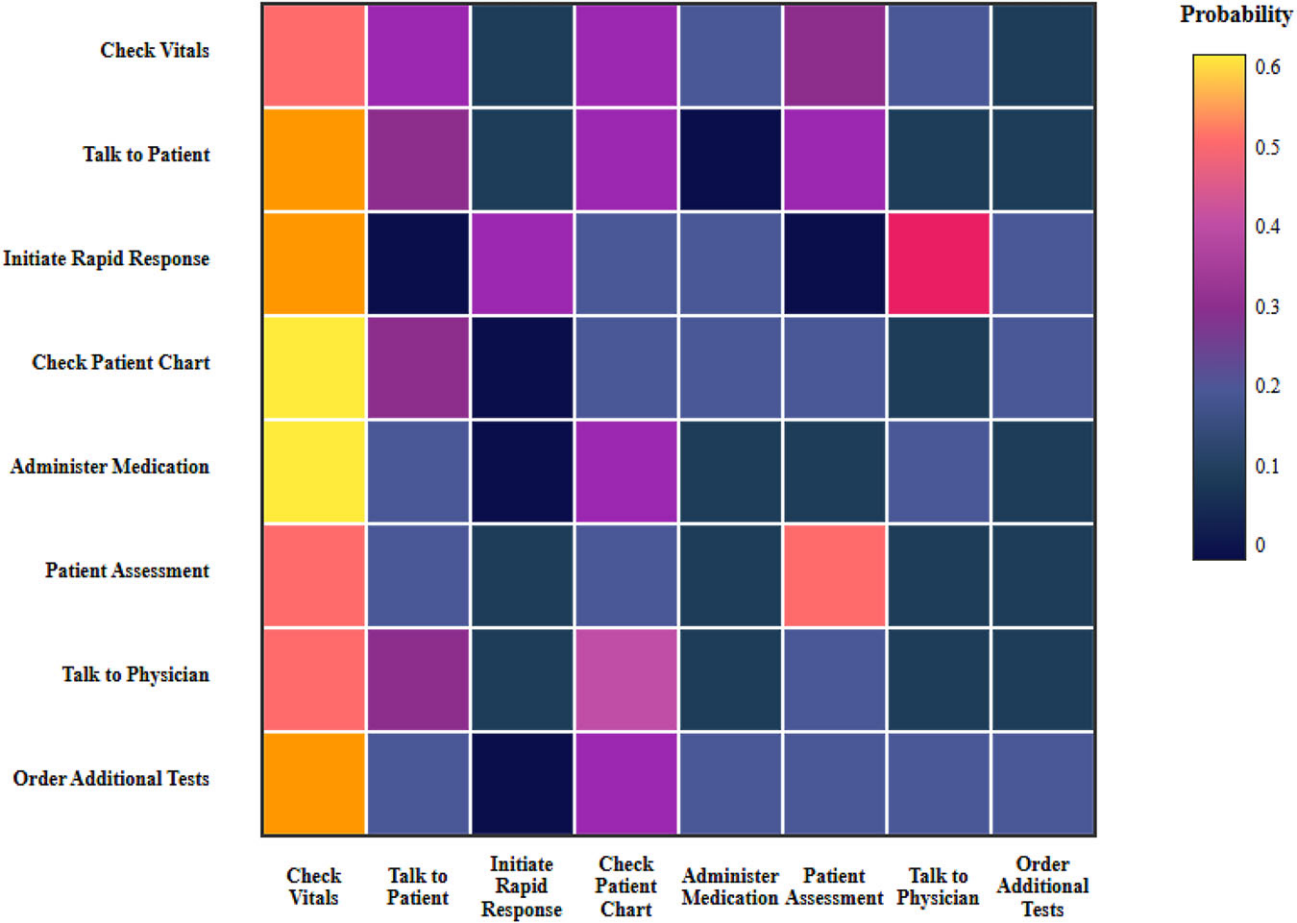
Transition probability matrix. This figure shows the probability of each action following another action, where the rows represent the first action and the columns represent the second action (each row equals 100%).

During the stroke scenario, experienced nurses performed an average of 29.7 ± 11.7 patient care actions, while performing an average of 36.6 ± 8.7 actions during the Covid simulation. Nursing students performed 27.7 ± 10.6 actions during the stroke scenario (complete dataset composition details are detailed in Table 1). The initial analysis of both scenarios revealed that nurses performed 19 unique types of patient care actions during patient care simulations. These 19 actions were distilled into 8 common actions during the second task analysis phase. During the stroke simulation, nurses and students checked vitals (i.e., attending to the vital sign monitor) most frequently (37.1%), followed by performing a focused patient assessment (e.g., checking patient pulses or performing a neurological assessment) (20.5%), and talking to the patient (16.2%) (Table 2). During the Covid-19 simulation, nurses checked vital signs most often (41.8%), followed by checking the patient chart (20.1%), and administering medication (11.1%) (Table 3). The overall distribution of data is shown in Fig. 1, highlighting the imbalance in actions. The patient care action performed with the highest frequency is to “check vital signs”, which is around 20% higher than the next most common action performed. We also plot a transition probability matrix, which describes the probability of one action following another, shown as a heatmap in Fig. 2. As expected from the frequency distribution, we see that the “check vital signs” action has a uniformly high probability of succeeding any action relative to its peers. We also noted two outliers. The “focused patient assessment” action was repeated at a higher frequency than other actions, and “initiate rapid response” was performed in conjunction with “talk to physician”.

**Table 1:** Dataset composition

Frequency statistics					
Participants	*n* = 24 Total	*n* = 13 Students		*n* = 11 Experienced nurses	
Episodes	*n* = 33 Total	*n* = 23 Stroke episodes (13 students, 10 experienced nurses)		*n* = 10 Covid episodes (0 students, 10 experts)	
Total actions	*n* = 1024 Total	*n* = 658 Stroke actions (361 students, 297 experienced nurses)		*n* = 366 Covid actions (all experienced nurses)	
Action sequence length statistics					
Scenario	Mean	Median	Standard deviation	Range	Interquartile range
All episodes	31	31	11	12–58	23–35
Stroke episodes (all)	28.6	25	11	12–49	20.5–34
Student stroke episodes	27.7	25	10.6	12–45	20–34
Experienced nurse stroke episodes	29.7	27	11.7	16–49	21.25–31.75
Covid episodes	36.6	34	8.7	25–58	31–41
Class imbalance summary					
Condition: 69.6% Stroke vs. 30.4% Covid actions			Group: 35.3% Student vs. 64.7% experienced nurse actions

**Table 2:** Percentage of patient care actions performed during the stroke simulation

Behavior	Percentage of behaviors performed
1 = Check vital signs	37.1%
2 = Talk to patient	16.2%
3 = Initiate stroke alert/rapid response	3.1%
4 = Check patient chart	12.8%
5 = Administers medication	2.3%
6 = Focused patient assessment	20.5%
7 = Talk to physician	6%
8 = Orders/administers additional diagnostic tests or medications	2%

Data includes experienced nurses and nursing students.

**Table 3:** Percentage of patient care actions performed during Covid-19 simulation

Behavior	Percentage of behaviors performed
1 = Check vital signs	41.8%
2 = Talk to patient	9.3%
3 = Initiate stroke alert/rapid response	0%
4 = Check patient chart	20.8%
5 = Administers medication	10.7%
6 = Focused patient assessment	8.2%
7 = Talk to physician	4.1%
8 = Orders/administers additional diagnostic tests or medications	5.1%

Data includes only experienced nurses, as nursing students did not complete the Covid-19 simulation.

**Fig. 3:**
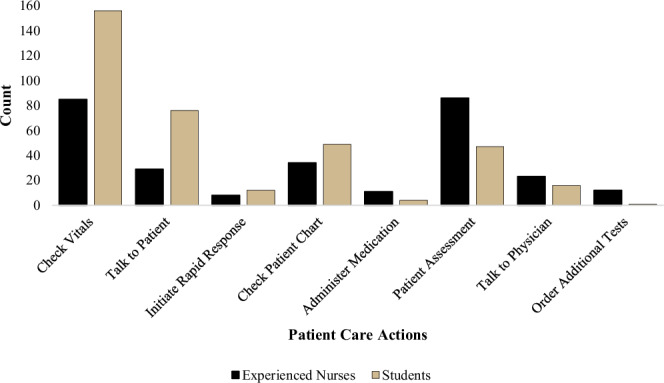
Experienced nurse and student patient care actions during stroke scenario. This figure shows the comparative distribution of actions performed by experienced nurses and students during the stroke scenario.

However, we did identify some variability in the dataset when comparing the cumulative dataset of experienced nurse behaviors during the stroke scenario to nursing students (Fig. 3). Unlike experienced nurses who had a balance between checking the vital sign monitor and performing focused patient assessments, nursing students relied heavily on the vital sign monitor for their information. The experienced nurses also administered medication and performed additional diagnostic tests proportionally more often than nursing students. These observed differences in patient care actions illustrate that our model was trained using variable behavior trajectories, which increases the generalizability of the model to effectively predict nursing actions when caring for ischemic stroke patients.

### Model performance

**Fig. 4:**
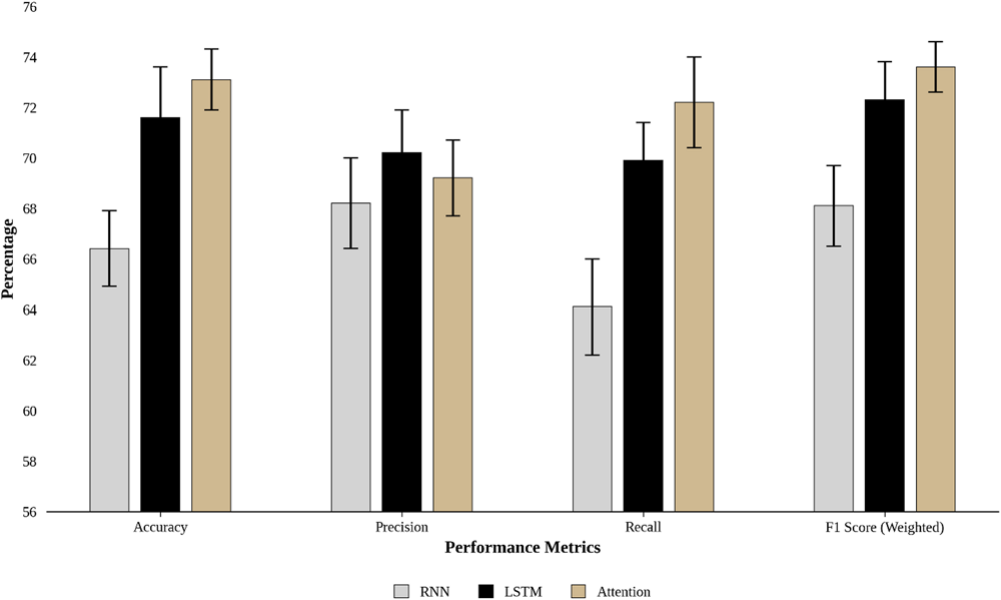
Model performance comparison. This figure shows the comparison of RNN, LSTM, and attention models with our data set based on accuracy, precision, recall, and weighted F1 score. Error bars indicate the standard deviation over the k-fold validation.

**Fig. 5:**
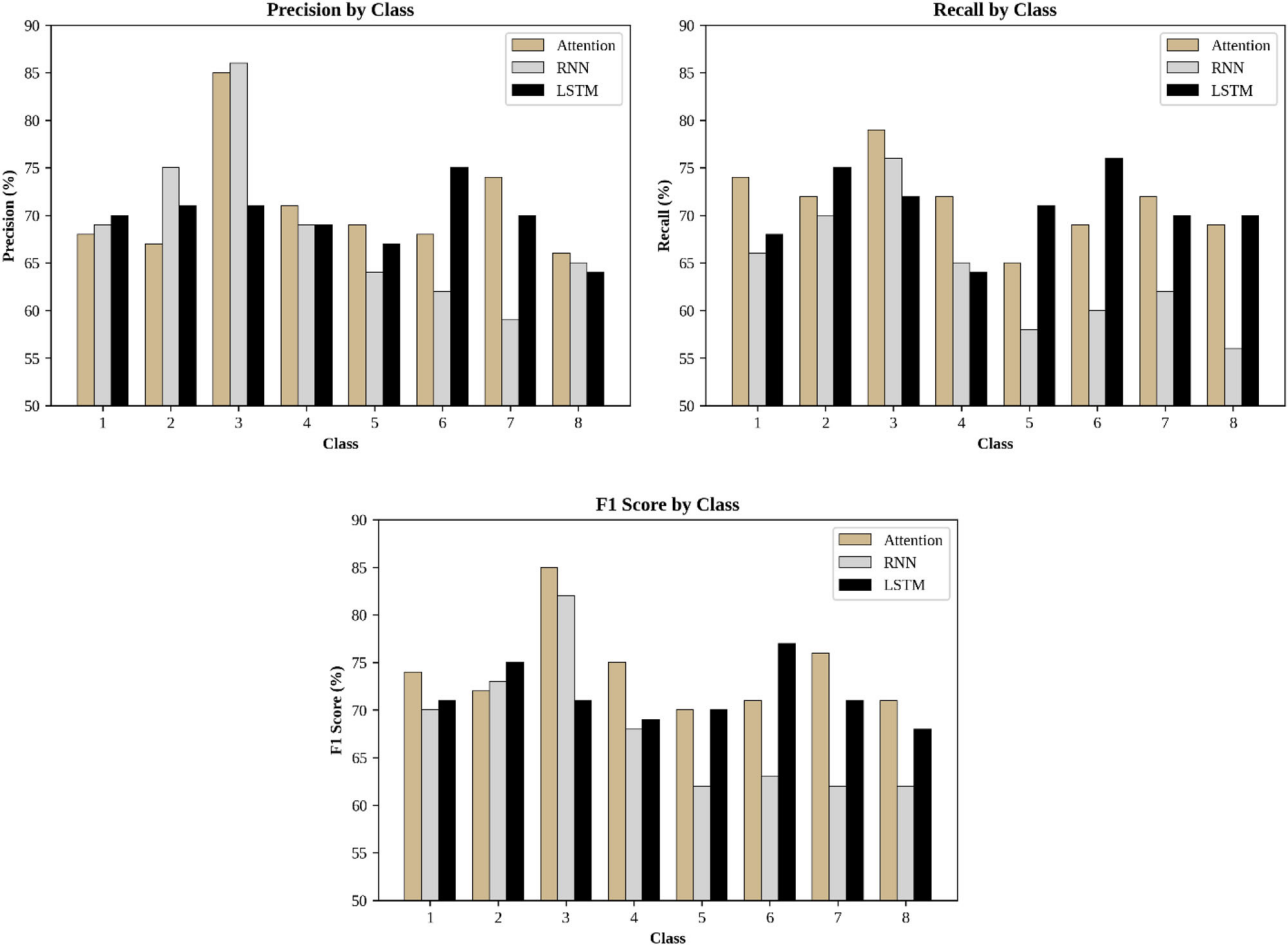
Per-class performance model comparison. This figure shows the comparison of Attention, RNN, and LSTM model performance per class based on precision, recall, and F1 score.

Overall, the behavioral prediction models used in our study were effective. All models outperformed chance selection of appropriate behaviors (i.e., maximum of ~60% probability of transitioning between actions, as evidenced in Fig. 2). The LSTM and attention models, specifically, displayed accuracy above 70%. When comparing the performance of the attention architecture to the RNN and LSTM methods with our dataset, we found that the attention architecture outperformed the others on accuracy, recall, and F1 score (Fig. 4). The LSTM model outperformed others in regards to precision. When analyzing the per-class performance between models (Fig. 5), we found that the attention model performed most consistently well. Regarding precision, the attention model had the highest score for 4 of 8 classes (i.e., compared to 3 of 8 for RNN and 1 of 8 for the LSTM model). In regards to recall, the attention model again had the highest score for 4 of 8 classes (i.e., the same as the LSTM model). Finally, the attention model had the highest F1 score for 5 of 8 classes (i.e., compared to 3 of 8 for the LSTM model).

Formal statistical comparisons between models using paired t-tests across the five cross-validation folds revealed that both LSTM and attention models significantly outperformed the baseline RNN model (LSTM vs. RNN: *t*(4) = 6.295, *p* = 0.003, mean difference = 5.42 points, Cohen’s *d* = 2.815; attention vs. RNN: *t*(4) = 11.089, *p* = 0.0004, mean difference = 5.78 points, Cohen’s *d* = 4.959). However, despite the attention model achieving a numerically higher mean F1 score than LSTM (0.37 point advantage), this difference was not statistically significant (*t*(4) = 0.812, *p* = 0.463, Cohen’s *d* = 0.363).

## Discussion

The purpose of this study was to demonstrate the utility of applying an attention-based encoder to model sequential nursing patient care actions and predict nurse actions when caring for patients in the simulated clinical environment. While researchers have begun applying deep neural networks to measure human actions in activities outside of healthcare, little research has focused on developing methods to model sequential nursing actions^26^. Overall, our study demonstrated the potential feasibility of applying attention-based ML architecture to model sequential nurse action data. All deep learning models evaluated in our study (i.e., attention architecture, RNN, and LSTM) outperformed chance selection of appropriate actions. For example, if the models exclusively recommended checking the patient chart after checking the vital sign monitor, they would have had an accuracy of around 60% (Fig. 2). Indeed, all models had an accuracy above 60%, indicating more nuanced action prediction. The attention model had an accuracy of 73%, indicating appreciably better accuracy than chance probability. These findings are significant contributions to the field as they demonstrate that deep learning models, like the attention-based encoder, have the potential to accurately predict nursing actions from sequential action data.

The results from our study provide insights into sequential nursing care actions in complex adaptive systems. First, we found that when comparing nurse actions when caring for ischemic stroke patients and patients suffering from Covid-19, there are differences in the frequency nurses check the patient chart, administer medications, and perform focused patient assessments. These differences may reflect scenario-specific requirements in nursing approaches that were adopted in this study. The multi-patient nature of the Covid-19 scenario meant that nurses had to recall additional patient background information, and thus, had to refer to the patient chart more frequently to support information recall. Also, nurses were forced to adjust oxygen treatments (i.e., administer medications) for patients during the Covid-19 scenario, but the stroke scenario did not require this action. Finally, the focused patient assessment differences between cases can be attributed to nurses’ need to assess multiple patient systems (e.g., neurological status, pulses, asymmetrical weakness) to confirm the possibility of a stroke during that scenario. Otherwise, the distribution of nurse actions was relatively consistent between cases, which suggests that sequential nurse action data may be largely generalizable.

In regards to transitions between actions, we found that following every initial action, nurses had the highest probability of transitioning to checking the vital sign monitor. This sequence of actions may highlight nurses’ use of quantitative data to corroborate qualitative data information gathering approaches (e.g., talking to the patient and performing focused patient assessments). We also found that nurses routinely talked to patients to gather information following checking vital signs and consulting the patient chart, which suggests that gathering patient state data directly from the patients supports their care process. Research has previously shown that experienced nurses frequently confer with patients and their families to develop a holistic picture about how a patients’ state has evolved from baseline^8^. Findings from the current study support this contention. Finally, we found that nurses had a high probability of performing additional focused patient assessments after an initial patient assessment. The literature emphasizes the need for nurses to conduct physical patient assessments to gather comprehensive insights about a patient’s status and identify abnormalities^27^. The high probability of repeated focused patient assessments observed in the current study represents the importance of complete physical examinations in the nursing care process.

One benefit of modeling sequential nurse patient care actions and making recommendations is the preservation of nurses’ holistic information-gathering methods. There are currently offline RL-driven systems designed to interpret quantitative data and provide diagnostic assistance for healthcare providers^15^. However, these systems face potential limitations due to their inability to capture qualitative data that a human nurse can capture (e.g., speaking with the patient about how their symptoms have evolved). Our proposed approach to process sequential action data can overcome this limitation by making informed recommendations on patient care actions that incorporate quantitative and qualitative sources of patient data. In instances where patients are suffering from disease processes that require triangulation of data sources to make correct diagnoses (e.g., verifying patients are suffering from ischemic stroke through vital sign monitoring and assessment of neurological symptoms), this sequential task modeling approach may be more supportive of nurse decision-making than diagnostic suggestions alone. The comparison of DM outcomes between traditional diagnostic support tools and our proposed approach warrants further investigation in the future.

In comparison to the accuracy, recall, and F1 score of LSTM and RNN, the successful implementation of the attention architecture used in our study should also be encouraging for ML researchers. In regards to behavioral research of healthcare providers, data is often extremely limited by small sample sizes^28^. Research in the clinical environment is subject to numerous barriers, including a lack of standardization of disease pathologies and staff cross-coverage of patients, among numerous other limitations^29,30^. Patient care simulations offer a more standardized and controlled opportunity for research^31^. However, due to logistic limitations requiring busy healthcare staff to participate in studies after their clinical duties, it is difficult to accrue large sample sizes. The attention architecture used to model task sequences in the current study displayed more accuracy than other ML approaches with the same limitations in sample size. We also found that the attention model displayed more consistent per-class performance than the other models. Despite the attention-based model displaying largely superior performance than other models for most metrics, we did observe that the LSTM model had better overall precision than the attention model and equivalent per-class recall with the attention model. We did find that the attention and LSTM models outperformed the RNN model statistically, but there were no statistically meaningful differences between the attention and LSTM models. The literature shows that attention models often increase recall on minority classes by being more sensitive to rare data patterns^32^. That being said, model precision can drop appreciably if they over-predict minority classes^33^. Furthermore, LSTM models may be more conservative than attention models and favor majority classes, which boosts precision while lowering recall^34^. Given the limited amount of data in our study and the high variability in per-class support, we may have been unable to detect meaningful statistical differences between LSTM and attention models. However, despite a lack of statistical differences, the attention-based model used in our study appears to be an appropriate method to evaluate and predict sequential patient care actions, given its high per-class consistency and overall performance.

There were limitations in our study. The dataset used in this study was relatively small and may have had inherent biases. Working with limited data is a common challenge in healthcare analytics that can lead to overfitting and poor generalization^35^. To mitigate these risks, we employed several key techniques. First, we used k-fold cross-validation for training, which allowed us to evaluate the model on all examples while making efficient use of the limited data. We also found that there was a class imbalance, with some nursing behaviors being significantly underrepresented compared to others. To avoid biasing the model towards frequent majority classes, we weighted the loss contribution of underrepresented classes higher during training. Finally, we employed regularization techniques to restrict the model’s capacity and prevent overfitting to the small dataset’s peculiarities. Even with these precautions, any potential biases present in the data collection process need to be investigated thoroughly. It is also possible that our convenience sample of experienced nurses and voluntary pool of nursing students introduced selection bias in our sample, as data may not be representative of patient care actions performed by nurses and students in general. This bias could ultimately limit the generalizability of our findings. Unfortunately, given the demands imposed on participants to complete the simulations, random sampling was not feasible for our study. In the future, soliciting participation from nurses and students from other hospitals and academic institutions could improve the generalizability of our findings.

One theoretical limitation in our approach to model nurses’ patient care actions is privacy concerns related to capturing video of nurses’ completion of care tasks. While the current study did not face privacy concerns given our adherence to IRB standards of maintaining participants’ confidentiality and data collection in the simulated environment, deployment of our model in the clinical environment may face patient privacy concerns related to protected health information. Accordingly, studies aiming to reproduce this study should consider collecting additional nurse patient care action data in the simulated environment to avoid privacy concerns. Furthermore, this modeling approach for nurse action prediction should be deployed in the simulated environment as an educational aim in the short term before considering deploying similar technology in clinical settings. Another potential limitation in our study is the use of traditional ML model evaluation metrics with variable behavior trajectory data. Given the potential variation in the behaviors nurses performed throughout the scenario, it is possible that this would have contributed to a lower true positive rate for our model. To address this, our team intentionally classified diverse nurse behaviors into comprehensive categories. This approach (i.e., reducing 19 possible actions to 8 categories) has reduced the potential variability in trajectories resulting from similar nurse actions categorized as different behaviors (e.g., “assesses patient facial asymmetry” and “assesses pupils” are both elements of stroke assessment and are better captured by the category “performs focused patient assessment”) and enabled us to have confidence in the true positive rates we observed. Finally, our model of nurse behaviors was developed based on data from ischemic stroke simulations and the generalizability of this model to other disease processes is not established, which is a limitation of our work. While we did attempt to enhance generalizability by incorporating variable action trajectories from nurses caring for Covid-19 patients, additional variable trajectories are needed to improve the generalizability of our model and expand it beyond ischemic stroke. The inclusion of more variable trajectory sequences can limit attention models’ memorization of specific actions and learn underlying action structures instead (e.g., spatial-temporal interactions), as evidenced in autonomous vehicle research utilizing multi-modal data for attention model training^36^. That being said, the presented approach of modeling sequential nurse-patient care actions using attention architecture is expected to be generalizable.

In the future, our team plans to extend this research in several ways. First, our team is presently collecting experienced nurses’ and nursing students’ patient care action data during two novel patient care simulations (i.e., respiratory and cardiac failure). This additional data will be used to train and validate our model to enhance its generalizability. Furthermore, in order to support the integration of our nursing action predictive model with clinical decision support tools, our team will revise our model and pair simulated patient diagnostic trajectories with nursing action data to create a more comprehensive approach to decision support. Then, in a simulated patient care setting, our team will evaluate the relative impact of the novel two-pronged approach to decision support compared to existing clinical decision support algorithms to evaluate differences in nurses’ escalation of patient care protocols. We anticipate the near future benefits of our system are educational, as nurse educators may be able to leverage our system to support nursing students caring for ischemic stroke patients in the simulated setting. For example, with documentation of student actions in real time, the system could automatically recommend the next action to be performed by students in the event they face difficulty. Determining the impact of our system on educational outcomes (i.e., compared to standard pre-simulation didactic training) for nursing students is another important area of future research for our team. These future steps will allow us to address generalizability concerns through expansion of our data set with multiple disease processes and nurse patient care action trajectories, patient privacy concerns by conducting additional data collection in the simulated patient care setting, and human factors considerations by determining how this system can support nursing students’ cognitive factors (e.g., situation awareness and decision making) when caring for critically ill patients in the simulated environment. environment and how this system impacts nursing educational outcomes.

## Methods

### IRB statement

Regulatory approval for this study, which adheres to the ethical standards detailed in the Declaration of Helsinki, Belmont Report, and the United States Common Rule, was provided by the Purdue University Institutional Review Board (IRB# 2020-1684, 2021-1337). In accordance with ethical guidelines, written informed consent was obtained from all participants before completing any study-related procedures.

### Nurse patient care action data

Data were obtained from a convenience sample of experienced nurses who were known to the study team at the time of data collection and were recruited intentionally based on their clinical expertise. Nursing students were also recruited to participate in the study during a simulation training day for their nursing program. We aimed to recruit experienced nurses and students to obtain varied behavior trajectories; that is, we expected that students and experienced nurses would differ in the patient care actions they performed when caring for simulated patients, given their differences in clinical knowledge and skill. Experienced nurses engaged in two 10-min simulated patient care encounters, where they individually cared for simulated patients (i.e., SimMan 3G, Laerdal, Stavanger, Norway) in the emergency department. All scenarios were video recorded for analysis at a later date. Nurses were instructed to perform all actions they would normally perform in the clinical environment and to escalate patient care if needed. Before the simulations, nurses received a standardized introduction to the simulated space (e.g., the patient manikin’s capabilities and available equipment). At the start of each scenario, nurses were provided a standardized handoff from a member of the study team who described the patient’s status, background, assessment, and physician orders. Following the handoffs, nurses entered the patient’s room to begin initial assessments. In the first simulation, nurses cared for a patient suffering from a traumatic leg fracture resulting from a motor vehicle crash. Nurses were told the patient was scheduled to have surgery the next day. The patient was responsive and displayed normal vital signs for the initial phase of the scenario, which lasted for approximately five minutes for all participants. Following this initial assessment phase, the patient experienced the sudden onset of a stroke. The scenario continued until nurses ordered a rapid response team to care for the patient, or a period of five minutes elapsed. If the nurse did not order a rapid response team explicitly, they were asked by a nursing faculty member for their diagnosis of the presenting problem. In the second scenario, nurses evaluated two patients suffering from complications from suspected infection with the SARS-CoV-2 (Covid-19) virus. One patient was more critically ill than the other, as they were suffering from complications related to sepsis. After performing initial assessments and ordering laboratory tests, nurses were asked for their diagnosis of the critically ill patient. Nursing students only participated in the ischemic stroke simulation as part of their simulation-based education, which was the same scenario that experienced nurses completed.

### Task analysis process

After all testing sessions were completed, task analyses were performed by human factors and nursing experts to determine the specific actions and patterns of actions performed by nurses during all phases of the stroke and Covid-19 simulations. Task analysis is a technique utilized by human factors experts to systematically describe the actions performed by humans in complex systems to execute discrete tasks^37^. Video recordings from participants’ completion of each scenario were uploaded to a custom annotation software for systematic task analysis, which allowed members of the research team to document each sequential patient care action performed by every nurse and the time when the action took place (i.e., initial patient assessment, change in patient status). Actions were coded according to an inductive content analysis approach, where all unique patient care actions (i.e., across both scenarios) were documented and categorized for each nurse’s simulated performance by two members of the team. Upon consensus being reached, actions were reviewed by clinical and human factors experts^38^. Following the initial task analysis for both scenarios, a member of the study team then aggregated all actions into a single workbook and developed a consistent patient care action coding framework to distill behaviors into one of eight categories: check vital signs, talk to the patient, initiate rapid response/stroke recognition, check patient chart, administers medication, perform focused patient assessment, talk to the physician, and order/perform additional diagnostic tests (Table 4 shows the mapping of original 19 actions to the condensed list of 8 actions). Our approach followed established response class analysis approaches, where discrete behaviors that serve the same function are systematically grouped into a single response class^39^. The use of a single analyst to develop a consistent coding framework and condense behavioral categories is supported by the task analysis literature, which indicates that single analyst approaches to job analysis can reduce sources of inaccuracy and are appropriate to perform task analysis of procedural skills in medical education^40,41^. This data was used to train the task prediction model.

**Table 4:** Mapping original nurse patient care actions to condensed nurse actions

Original action category	Condensed action category
Check vital signs	Check vital signs
Initiate stroke alert/rapid response/stroke recognition	Initiate stroke alert/rapid response/stroke recognition
Assess patient pain	Perform focused patient assessment
Assess patient facial asymmetry	
Assess patient speech	
Assess patient neurological status	
Assess breath sounds	
Assess pupils	
Assess pulses	
Assess numbness/weakness	
Check patient chart	Check patient chart
Administer medication	Administer medication
Provide other treatment (e.g., neck brace)	
Talk to patient	Talk to patient
Calm patient down	
Talk to physician	Talk to physician
Consult neurology	
Order/perform additional diagnostic tests	Order/perform additional diagnostic tests
Request medication	

### Development of sequential action prediction model

To address the need to predict the next patient care action given a sequence of previous actions, we employed an attention-based encoder model inspired by the transformer architecture proposed by Vaswani et al.^23^. By allowing the model to attend to different positions of the input sequence (i.e., sequential nurse patient care actions) when encoding each action, it captured intricate patterns and dependencies more effectively, which potentially led to more accurate predictions of the next action. Furthermore, the attention-based encoder model’s ability to process sequences in parallel and make predictions based on partial information aligned well with the dynamic nature of nurses’ patient care actions. Recently, Fintz et al. developed a deep neural network model specifically to predict human behavior during a four-armed bandit selection task (i.e., selection of one door out of four possible doors to maximize the reward) and found that their approach outperformed other ML approaches in accurately predicting human behavior^26^.

**Fig. 6:**
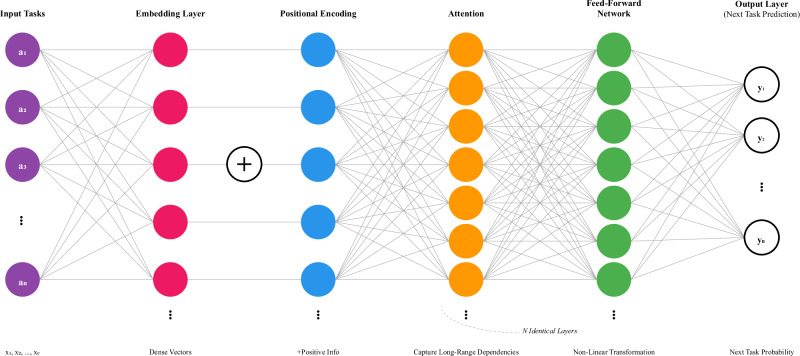
Attention-based nurse action prediction model. This figure shows the structure of our attention-based patient care action prediction model.

The developed model contained an embedding layer, a positional encoding layer, and a stack of transformer encoder layers (Fig. 6). The embedding layer mapped each action to a dense vector representation. The positional encoding layer introduced positional information to the embeddings, enabling the model to capture the order of actions in the sequence. The core of the model is the transformer encoder, which consists of *N* identical layers. Each layer consisted of two sub-layers: a multi-head (i.e., attention heads = 2) self-attention mechanism and a fully connected, position-wise, feed-forward network (i,e., Feed-forward dimensions = 32). The multi-head attention method enabled the model to attend to different portions of the input sequence when encoding a specific action, effectively capturing long-range dependencies. The feed-forward network applied non-linear transformations to the output of the attention mechanism, which further enriched the representation. The output of the transformer encoder was a sequence of encoded representations, one for each action in the input sequence, capturing the relationships between actions. We trained the attention-based encoder model using a language modeling approach, where the objective was to predict the next nurse task given a sequence of previous actions. The model’s output for each input sequence was then compared to the target sequence using the cross-entropy loss function. All simulation data, including student and experienced nurse stroke sequences and all Covid-19 sequences, were used for model training. Since data splits were at the subject level, a k-fold cross-validation approach was used to capture systematic differences, and the dataset was divided into *k* = 5 folds. The model was then trained 5 times, using a different fold for validation in each iteration while the remaining 4 folds formed the training set. Training was performed using the Adam optimizer and backpropagation to minimize the cross-entropy loss over the training data^42^. Regularization techniques were employed (i.e., early stopping and dropout) to mitigate overfitting and improve generalization^43^.

Actions were tokenized as categorical features using integer encoding for subtask values and binary encoding for assessment elements, condition, and participant group, with continuous features like timestamps normalized as needed. Variable-length sequences were handled through padding to a fixed maximum length, with masking applied during training to ensure padded positions did not contribute to loss calculation or attention mechanisms. Weighted loss derivation used inverse frequency weighting calculated as the ratio of total samples to the product of the number of classes and class-specific sample count. The dataset exhibited multiple nested imbalances across scenarios, nurse actions, and participant groups that required weighting. Nurse action imbalance was particularly extreme, so rare classes received substantially higher weights than dominant classes. In order to assess the effectiveness of our model, validation metrics were compared between the attention-based nurse action prediction model and a recurrent neural network (RNN) and a long short-term memory (LSTM) approach. Parameters of all models are included in Table 5.

**Table 5:** Model parameters

Parameter	RNN	LSTM	Attention
Input size	16	16	16
Hidden size	32	24	16
Number of layers	2	1	N
Output size	16	16	16
Total parameters	~3200	~3700	~3300
Dropout rate	0.1	0.1	0.1
Epochs	5	10	10

Data include all parameters utilized across the three ML models compared in the current study. Early stopping was used to prevent overfitting.

### Validation

Considering our imbalanced data classes, we used the precision, recall, and F1 score metrics for evaluation. These metrics are crucial for imbalanced datasets where some classes significantly outnumber others, as traditional accuracy can be misleading by overemphasizing the correct predictions of the majority classes. Precision and recall focus on the model’s performance in identifying positive instances and capturing all relevant positive instances, respectively. The F1 score balances these aspects, providing a comprehensive evaluation of the model’s effectiveness in handling the minority classes. Finally, we compared models statistically using paired t-tests across the five cross-validation folds. *P* values less than 0.05 were considered statistically significant.

## Data Availability

The datasets generated and/or analyzed during the current study are not publicly available to ensure regulatory compliance, but are available from the corresponding author on reasonable request.

## Code availability

The underlying code for this study [and training/validation datasets] is not publicly available but may be made available to qualified researchers on reasonable request from the corresponding author.
